# 

**Published:** 1892-11-12

**Authors:** 


**Notices of Births, Deaths, and Marriages,** are inserted in this column at a charge of 2s. 6d. for an announcement not exceeding four lines.

### NOTICE TO CORRESPONDENTS.

All MS., Letters, Books for Review, and other matters intended for the Editor should be addressed The Editor, The Lodge, Porchester Square, London, W.

The Editor cannot undertake to return rejected M.S., even when accompanied by stamped directed envelope.

### Notice to Advertisers and Subscribers.

All Advertisements, Orders for Copies of The Hospital, and Business Communications should be addressed to The Publisher, *not to the Editor*, The Hospital, 140, Strand, W.C.

The Cost of Subscription, post free, is as follows:—Per week, 2½d.; per quarter, 2s. 6d.; per half-year, 5s.; per annum, 8s. 6d. Foreign:—Per annum, 10s. 8d.; payable, in all cases, in advance.

### "Burdett's Hospital Annual," 1891—1892.

Third year of publication. 600 pp. Boards, gilt lettering. A most complete guide to British and Colonial Hospitals, Dispensaries, Nursing and Convalescent Institutions. and Asylums. Price 3s. 6d. Published by The Scientific Press (Limited), 140, Strand, W.C.

**NOTICE.—The Index for Vol. XII. (ending September, 1892) is on sale at the Office of this Journal, price 2½d., post free.**

### SCRAPS AND GLEANINGS.

An anonymous friend has sent a donation of one thousand pounds to the Royal Medical Benevolent College.

The first cheque entered into the "Kate Marsden Leper Fund" was a donation from the Princess May of Teck.

The Royal Hospital for Diseases of the Chest, City Road, has received a grant of £100 from the Corporation of London.

The Queen has approved the appointment of W. C. M'Vail, M.D., as Crown Member of the General Medical Council for Scotland.

At a recent meeting of the Midland branch of the British Dental Association, it was proposed to establish a dental hospital or school in Sheffield.

The Governors of the Royal Holloway College, Egham, have appointed Mr. H. L. Callendar, Fellow of Trinity College, Cambridge, to the Professorship of Physics.

The Board of Management of the Chesterfield Hospital have decided to add a new wing to the present building. The Duke of Devonshire has given £500 towards the building fund.

The late Mr. Thomas Nelson, publisher, has left £50,000 for the erection of five working men's clubs and reading rooms. Among other institutions to receive benefits is the Edinburgh Infirmary.

Harold Stephenson, aged 11, the son of Mr. A. F. Stephenson, of Southport, has died from the effects of a kick on the thigh while playing football in the grounds at Brookfield School, Wigton.

It is reported that the King of Italy has promised to open the International Medical Congress, which is to be held in Rome in 1893. The inauguration of the Congress has been fixed for September 24th.

The Lord Mayor-Elect intends, with the sanction of the Court of Common Council, to give an entertainment at the Guildhall on November 11th to about 2,000 children belonging to the Board schools of the City.

During the past month the Royal Humane Society awarded medals and testimonials to ten young persons (whose ages averaged fourteen years each) for saving life from drowning in various parts of the kingdom.

A very successful theatrical entertainment in aid of the Richmond Convalescent Home, Worthing, was given by the Worthing Amateur Dramatic Society, which resulted in the sum of £17 2s. 10d. being handed over to the Home.

The number of new patients treated at the Ramsgate and St. Lawrence Royal Dispensary during the year was 1,937, against 1,894 in the previous year. The committee acknowledged at their annual meeting a legacy of £804 9s. 2d. from the late Mr. W. B. Good.

At the first annual meeting of the Wellington and District Cottage Hospital it was stated that during the three months the hospital has been open the whole of the six beds have been almost constantly occupied. Miss Dallas has presented an accident bed to the hospital.

Towards the Victoria Home for Invalid Children, which is to be established at Margate, Princess May has undertaken to collect £100. The Princess will receive all subscriptions sent to White Lodge, Richmond Park, and will particularly welcome donations, however small, from children.

At the half-yearly meeting of the Bolton Hospital Saturday Committee it was stated that the total amount collected for the year amounted to £1,822. From March 1st 600 new patients have been treated at the infirmary, an increase of 95 as compared with the same period last year.

Several students of Alston College, near Preston, went last week for a run across country. Two of them, named Young and Pool, were crossing the Ribble by jumping from rock to rock, when one stumbled and fell into a pool twenty-five feet deep, dragging the other with him. Both were drowned.

At the seventy-fifth annual meeting of the Birmingham Royal Orthopædic and Spinal Hospital the report was read, showing that the number of attendances in 1892 was 6,425, compared with 6,209 in 1891. The number of in-patients was exactly 200, and the average period of residence was 36 days.

An effort is being made by the vicar of St. Luke's Church, Victoria Docks, and a committee of social workers, to organise a series of free breakfasts for the dock workers and others, in the parish connected with the shipping trade, who are unable to procure work owing to the depression in the riverside work.

The local authorities at Dewsbury, after much discussion, have at last decided to apply to the Local Government Board for a provisional order for constituting a joint hospital scheme for the districts of Dewsbury, Heckmondwike, Soothill Nether, Soothill Upper, and Ravensthorpe to provide hospital accommodation for infectious cases.

The Select Committee on the Metropolitan Hospitals, in a report just issued, propose the formation of a Central Board which might be granted a charter to entitle it to receive endowments, legacies, bequests, and contributions for distribution to medical charities, and exercise a general control over auditing the accounts of these institutions.

Since the Hospital for Sick Children, Edinburgh, was first opened, upwards of 170,000 children have been treated in it. The directors have now decided to build a new hospital at a cost of between £25,000 and £30,000. Towards this object Lady Jane Dundas has given a donation of £10,000—£5,000 to be applied to the building and £5,000 to the endowment of one of the wings of the hospital.

The hospital vessel, "Albert," belonging to the Mission to the Deep Sea Fishermen, has returned to St. John's after a voyage of 75 days along the Labrador coast. The vessel visited altogether 50 different fishing settlements. Mr. W. T. Grenfell, the surgeon on board the "Albert," treated nine hundred patients among the fishermen, some serious cases, although only two deaths occurred. A large quantity of clothing was also distributed to those who required it.

### THE STUDENT OF MEDICINE.

### NOTES FROM THE SCHOOLS.

### St. Bartholomew's Hospital.

The entrance scholarships have been awarded as follows:—1. Open Science Scholarship in Chemistry and Physics, value £75, to Charles Todd, B.A., Clare Cottage, Cambridge. 2. Open Science Scholarship in Biology and Physiology, value £75, to Walter d'Este Emery, Mason's and Queen's Colleges, Birmingham. 3. Open Science Scholarship in Biology, Chemistry, and Physics, value £150, to Stanley Bean Atkinson, Prel. Sc., Lond. 4. Preliminary Scientific Exhibition in Biology, Chemistry, and Physics, value £50, to Joseph Henry Churchill, of City of London School. 5. Jeaffreson Exhibition in Classics and Mathematics, value £20, to James Hugh Thursfield, B.A., Trinity College, Oxon.

### Bristol Medical School.

The new medical wing of the University College, Bristol, will be opened by Sir Andrew Clarke, Bart., M.D., LL.D., F.R.S., on Wednesday, November 16th, 1892, at half-past four p.m. The annual dinner of the Bristol Medical School will be held at the Royal Hotel at seven for half-past seven p.m., Sir Andrew Clarke, Bart., in the chair.

### Edinburgh University.

Royal Medical Society.—The following have been elected presidents for the present session: A. N. S. Carmichael, M.B., C.M.; J. W. Crerar, M.B., C.M.; Lim Boon Keug, M.B., C.M., and Harry Rainy, M.A., M.B., C.M. The opening address of the session was delivered by Dr. Lauder Brunton, in the society's hall. The following have been appointed resident physicians to the Royal Infirmary: D. Macaulay, M.A., M.B., C.M.; D. C. Watson, M.B., C.M.; D. C. Edington, M.B., C.M.; J. Anderson, M.B., C.M.; J. S. Fowler, M.B., M.R.C.S.; R. O. Petril, M.A., M.B., C.M.; J. Baynes, L.R.C.P., L.R.C.S.E.; resident surgeons —C. A. Sturrock, M.A., M.B., C.M.; A. Duke, M.A., M.B., C.M.; G. C. Cathcart, M.B., C.M.; J. Maclaren, M.B., C.M.; A. K. Melville, M.B., L.R.C.S. The annual general meeting of the University Athletic Club was held on October 28th, Professor Annandale presiding. The number of members shows an increase of 59, and the accounts of the past year show a surplus of £80. Mr. W. D. Barron was elected honorary secretary, and the following were elected as committee: W. E. Fothergill, M.A., B.Sc.; G. St. C. Thom, J. H. A. Laing, M.B., C.M.; S. J. Aarons, J. G. C. Scott, J. M. Taylor, A. Steel, J. D. C. Howden.

### VACANCIES.

**The following vacancies are announced this week, the amount of salary in each case being given after the name of the institution:**

### Resident Medical Officers.

Birmingham General Dispensary, Resident Surgeon, doubly qualified—Salary £150 per annum, and £30 per annum allowance for cab hire. Applications to the Secretary by November 16th.

Borough of Bootle Hospital for Infectious Diseases, Resident Medica Officer, unmarried—Salary £100 per annum, with board, washing, and apartments. Applications to the Chairman of the Health Committee, Town Hall, Bootle, by November 8th.

Cancer Hospital (Free), Fulham Road, S.W., House Surgeon. Appointment for six months—Salary at the rate of £50 per annum, with board and residence. Applications to the Secretary by November 18th.

East London Hospital for Children, Glamis Road, Shadwell, E., House Physician; board and lodging provided. Applications to the Secretary by November 16th.

General Hospital, Barbadoes, West Indies, Junior House Surgeon—Salary £200 per annum, and quarters. Appointment for three years. Applications to the Secretary by December 1st.

Hospital for Sick Children, Great Ormond Street, W.C., House Surgeon, unmarried. Appointment for six months—Salary £25, with board and residence. Applications to the Secretary by November 15th.

Metropolitan Asylums Board, Assistant Medical Officer for the North-Eastern Hospital for Fever Patients, St. Ann's Road, Stamford Hill, N., doubly qualified—Salary £15 per month, with board, lodging, and washing. Applications to the Medical Superintendent at the Hospital.

Moulsford Lunatic Asylum, near Wallingford, Berks, Assistant Medical Officer—Salary £140 per annum to commence, rising £10 yearly to £160. Applications to Dr. Murdoch, Medical Superintendent, by November 10th.

New London County Asylum, Claybury, Woodford, Essex, Medica Superintendent, doubly qualified—Salary £1,000 per annum, with house, coals, lighting, milk, and vegetables. Applications on form to be obtained of Mr. R. W. Partridge, Clerk to the Asylum Committee, 21, Whitehall Place, S.W., by November 25th.

### The Hospital Advertiser.

### MEDICAL APPOINTMENTS.

**THE HOSPITAL FOR SICK CHILDREN,**
Great Ormond Street, W.C.

The Birmingham General Hospital,

**THE STUDENT OF MEDICINE—(*continued*).**

Paddington Infirmary, Assistant to the Medical Superintendent and Assistant Medical Officer of the Workhouse; unmarried; age between 23 and 30—Salary £100 per annum, rising £10 annually to £120, with board, lodging, and washing. Applications to H. F. Aveling, Clerk to the Guardians, 289, Harrow Road, by November 12th.

Royal Hospital for Incurables, Dublin, Resident Medical Officer, qualified in Medicine, Surgery, and to compound medicines—Salary £70 per annum, with board and furnished apartments. Appointment tenable for two years. Applications to Mr. Thomas Grey, Registrar. Election on November 14th.

Taunton and Somerset Hospital, Honorary Surgeon; Fellow, Member, or Licentiate of a Royal College of Surgeons, or Graduate in Surgery of a University in the United Kingdom, and duly registered. Applications must be received by November 21st. Must reside within two miles of hospital.

Smedley Hydropathic Establishment, Matlock, Derbyshire, Resident Physician; appointment for six months—Honorarium of 20 guineas. Board and residence free. Applications to the Secretary by November 5th.

Stafford County Asylum, Stafford, Junior Assistant Medical Officer; unmarried—Salary commencing at £100 per annum, with furnished apartments, board, and attendance. Applications to the Medical Superintendent.

Royal Infirmary of Edinburgh, Superintendent, must be member of medical profession—Salary £500 per annum, with free house and gas. Applications to Mr. James S. Trainer, Treasurer and Clerk, by December 5th.

Royal Westminster Ophthalmic Hospital, House Surgeon—Must be legally qualified and possess some knowledge of ophthalmic surgery; board and residence. Applications, with testimonials, on or before December 1st next. To take office January 1st, 1893.

Resident Medical Superintendent.

